# Supplementary material for: Characterize Oral-to-Blood Microbial DNA Translocation in Individuals with Cocaine Use Disorder
Source: bioRxiv. 2026 Jan 4:2025.11.03.686400. Originally published 2025 Nov 4. Preprint. [Version 3] doi: 10.1101/2025.11.03.686400 (PMC12637719; doi:10.1101/2025.11.03.686400)
Supplement: Supplement 1 [file NIHPP2025.11.03.686400v3-supplement-1.pdf]

bioRxiv preprint doi: https://doi.org/10.1101/2025.11.03.686400; this version posted January 4, 2026. The copyright holder has placed this preprint (which was not certified by peer review) in the Public Domain. It is no longer restricted by copyright. Anyone can legally share, reuse, remix, or adapt this material for any purpose without crediting the original authors.

Supplemental Figure 1. Box plots depict log2 fold changes in bacterial taxa between groups at the (A) phylum and (B) genus levels. Genus column median > 0.007 was selected.

Supplemental Figure 2. **Taxonomic composition of saliva and plasma microbiomes in CUD and controls.** Stacked bar plots of bacterial genera (A) and species (B) show the mean enrichment of oral- and plasma-associated taxa.

Supplemental Figure 3. **Effects of cocaine on *S. parasanguinis* growth.** Optical density (OD600) measurements of *S. parasanguinis* cultured under various conditions, including

bioRxiv preprint doi: https://doi.org/10.1101/2025.11.03.686400; this version posted January 4, 2026. The copyright holder has placed this preprint (which was not certified by peer review) in the Public Domain. It is no longer restricted by copyright. Anyone can legally share, reuse, remix, or adapt this material for any purpose without crediting the original authors.

media alone, media supplemented with cocaine (5 μg/mL and 50 μg/mL), and supplemented with glucose (100 mM).

Supplemental Figure 4. **Altered cell–cell communication and gene expression in PBMCs from CUD versus controls.** (A) Heatmaps show predicted incoming and outgoing signaling patterns across immune subsets, with CUD samples displaying distinct shifts in ligand–receptor signaling strength compared to controls. (B) Boxplots highlight representative DEGs, including *TSC22D3*, *CD83*, *CD300A*, *HLA-C*, *CAVIN2*, *GRAP2*, *EBX3*, *IFI16*, *DAP*, *LAIR2*, *FMN1*, *EPSTI1*, *RHOB*, *F2R*, *CDK11A*, *CD226*, *HLA-DQB1*, and *CEBPD*, demonstrating significant transcriptional alterations in CUD.

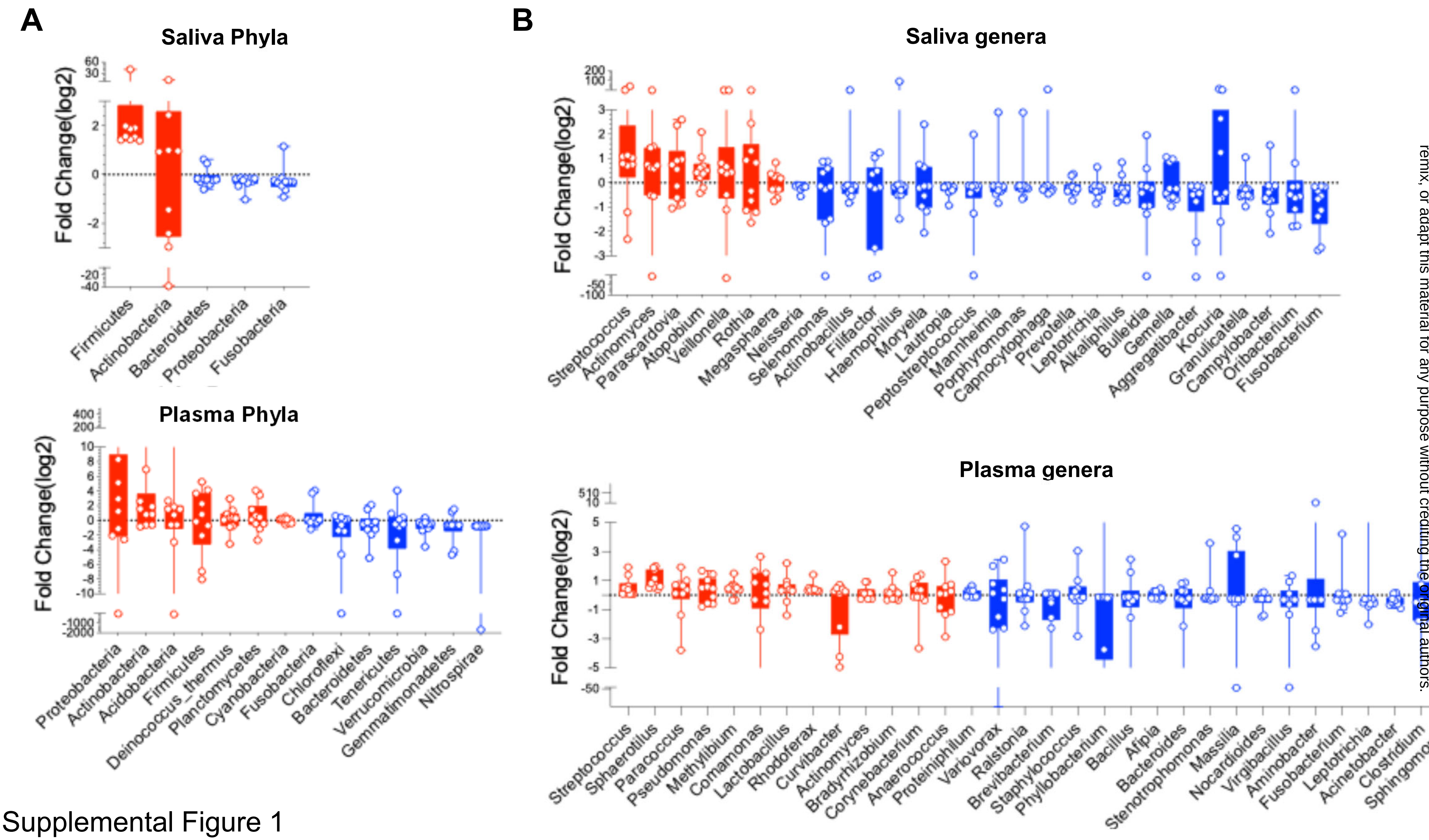

Supplemental Figure 1

bioRxiv preprint doi: https://doi.org/10.1101/2025.11.03.686400; this version posted January 4, 2026. The copyright holder has placed this preprint (which was not certified by peer review) in the Public Domain. It is no longer restricted by copyright. Anyone can legally share, reuse, remix, or adapt this material for any purpose without crediting the original authors.

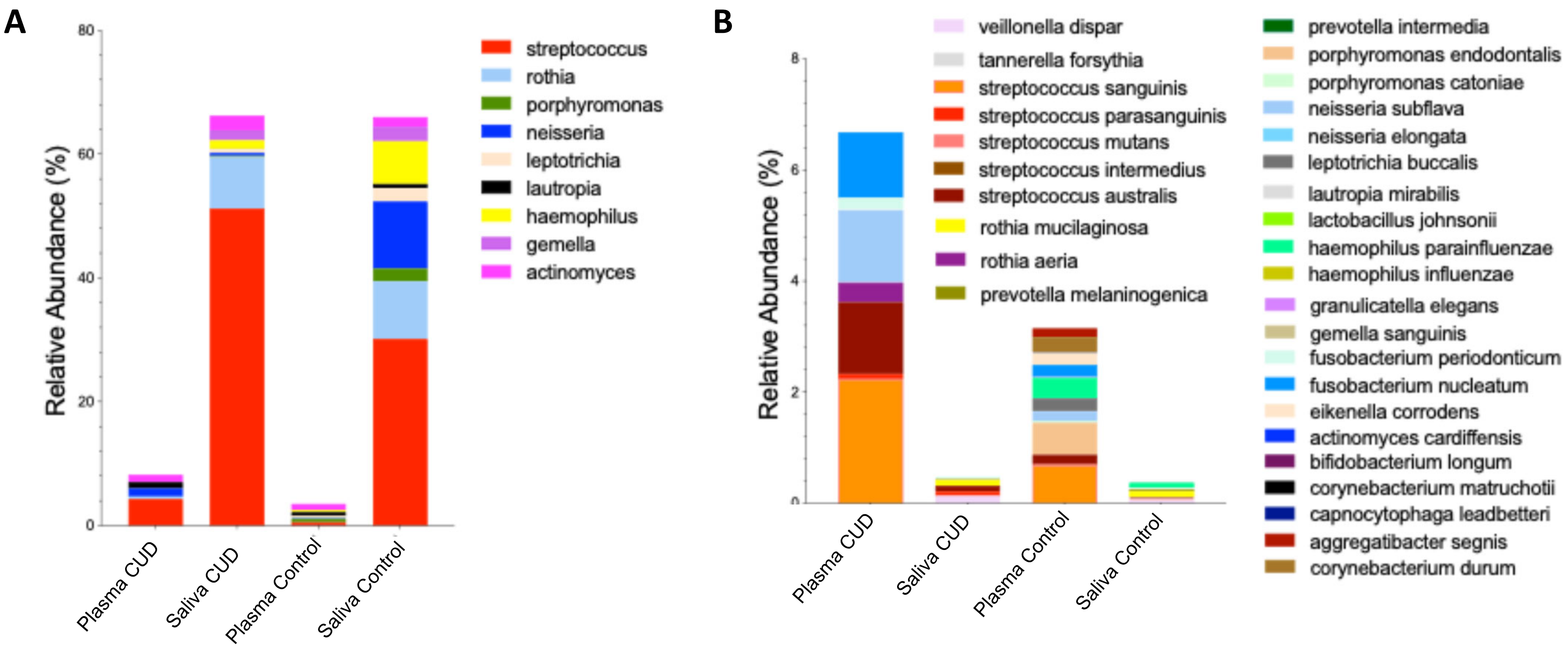

Supplemental Figure 2

bioRxiv preprint doi: https://doi.org/10.1101/2025.11.03.686400; this version posted January 4, 2026. The copyright holder has placed this preprint (which was not certified by peer review) in the Public Domain. It is no longer restricted by copyright. Anyone can legally share, reuse, remix, or adapt this material for any purpose without crediting the original authors.

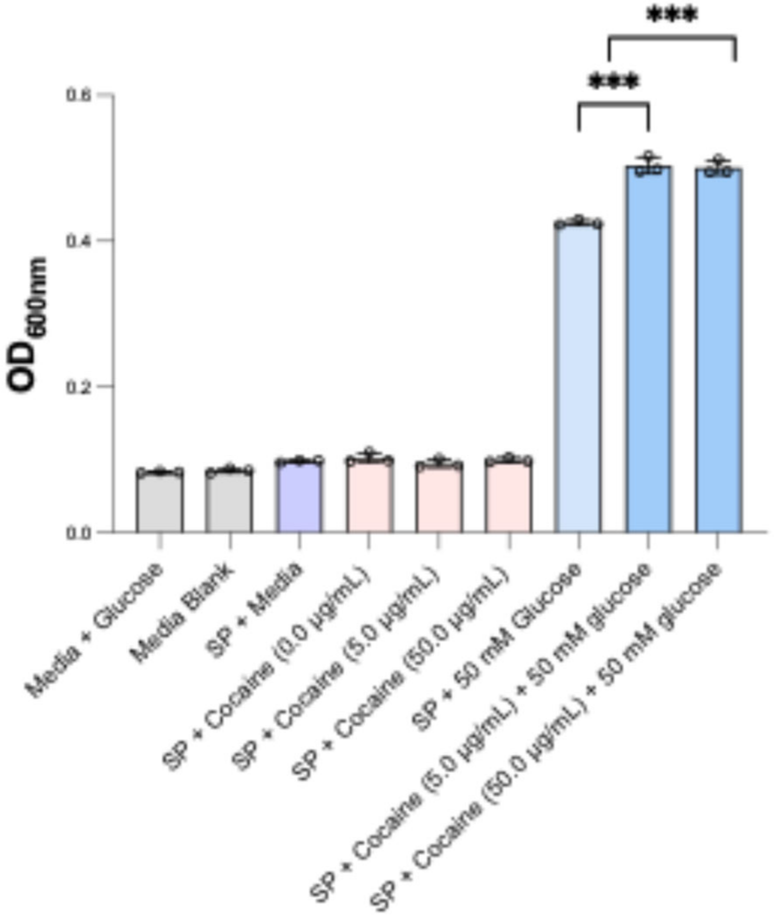

Supplemental Figure 3

bioRxiv preprint doi: https://doi.org/10.1101/2025.11.03.686400; this version posted January 4, 2026. The copyright holder has placed this preprint (which was not certified by peer review) in the Public Domain. It is no longer restricted by copyright. Anyone can legally share, reuse, remix, or adapt this material for any purpose without crediting the original authors.

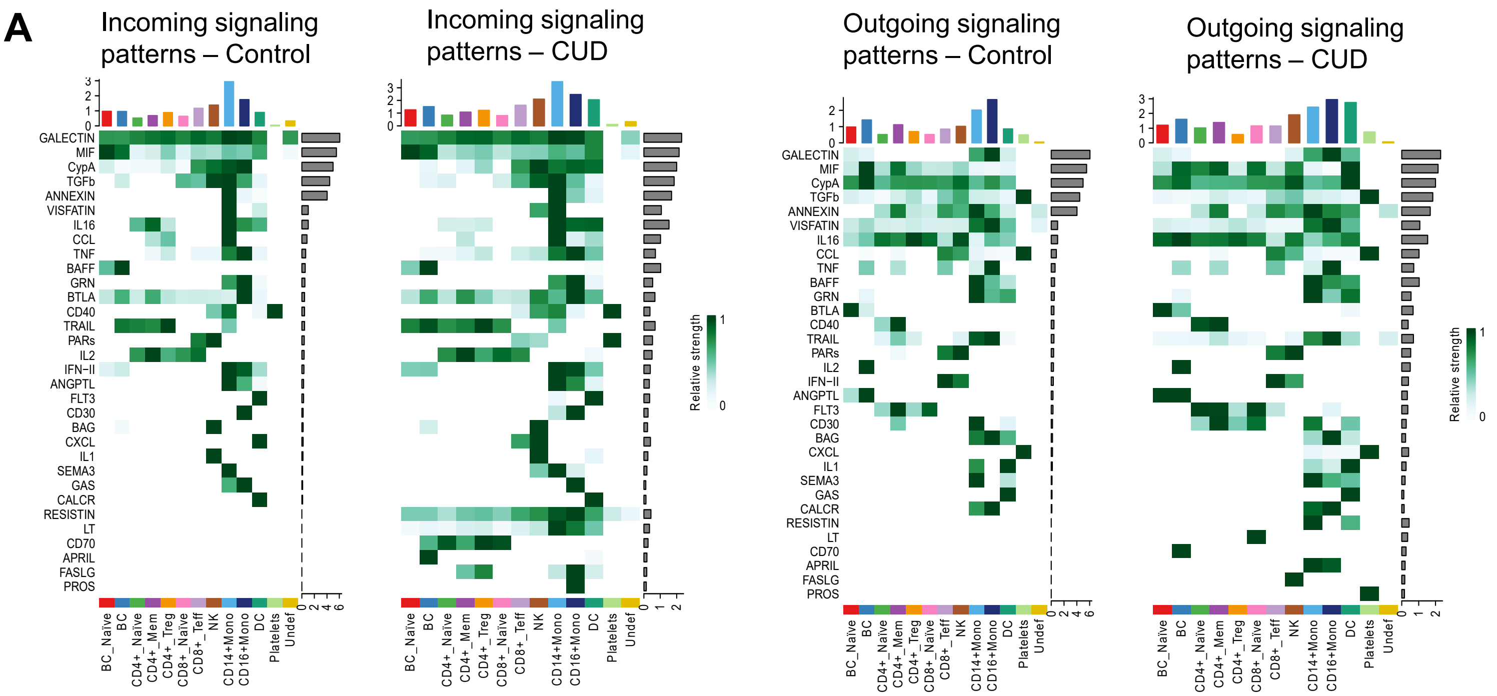

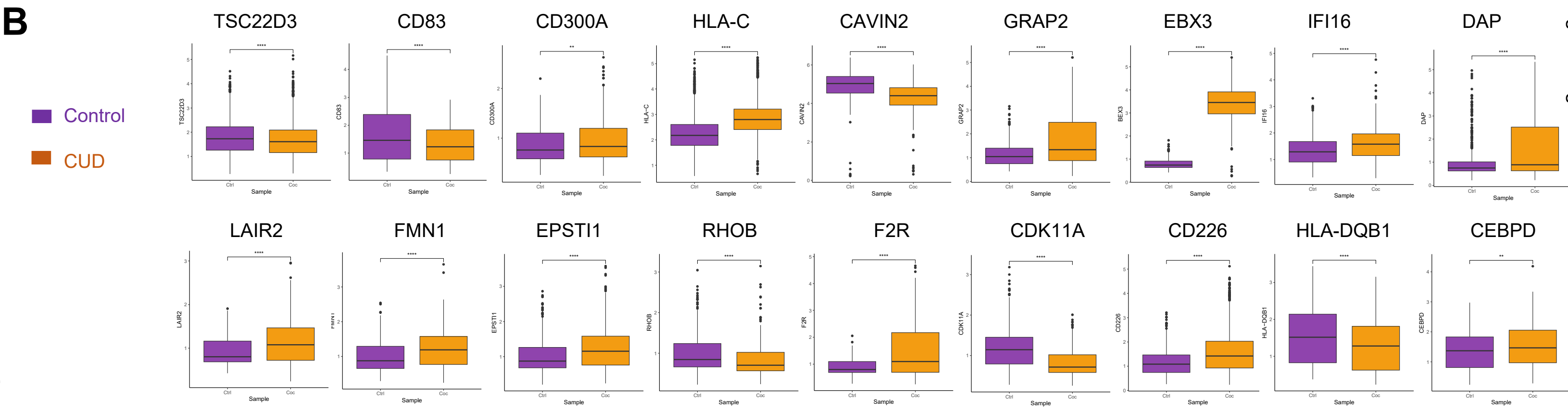

Supplemental Figure 4

bioRxiv preprint doi: https://doi.org/10.1101/2025.11.03.686400; this version posted January 4, 2026. The copyright holder has placed this preprint (which was not certified by peer review) in the Public Domain. It is no longer restricted by copyright. Anyone can legally share, reuse, remix, or adapt this material for any purpose without crediting the original authors.
