# Supplementary material for: Characterize Oral-to-Blood Microbial DNA Translocation in Individuals with Cocaine Use Disorder
Source: bioRxiv. 2025 Nov 4:2025.11.03.686400. Preprint. [Version 1] doi: 10.1101/2025.11.03.686400 (PMC12637719; doi:10.1101/2025.11.03.686400)
Supplement: Supplementary file 1 [file NIHPP2025.11.03.686400v1-supplement-1.pdf]

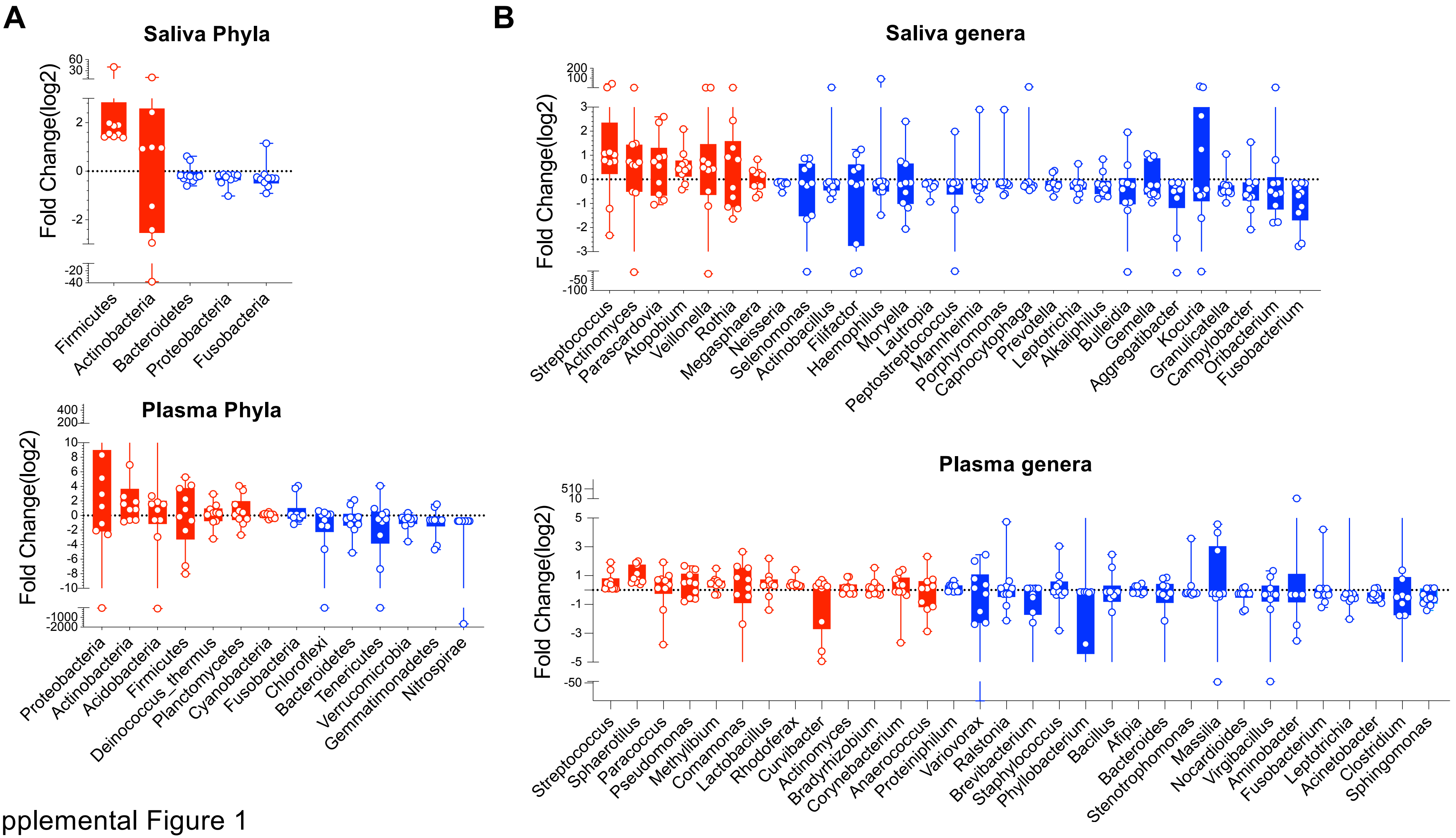

Supplemental Figure 1

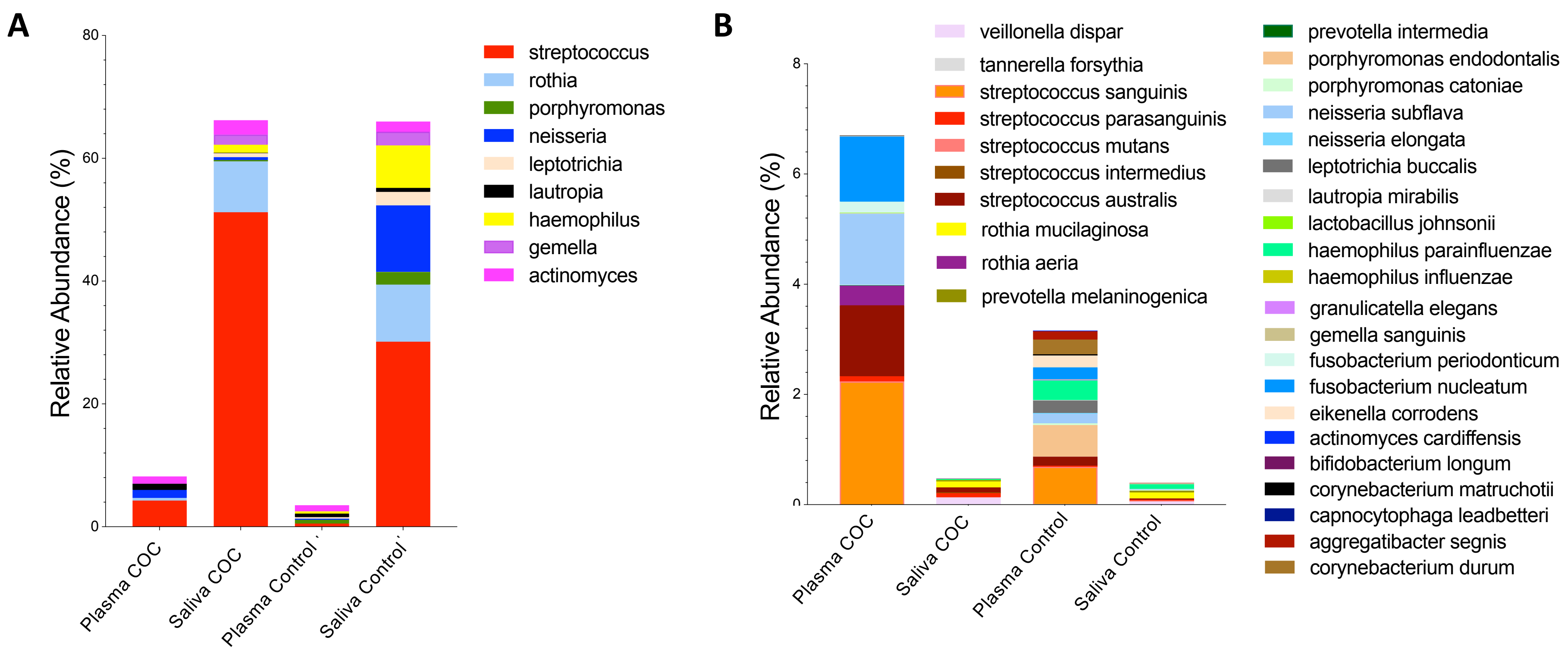

Supplemental Figure 2

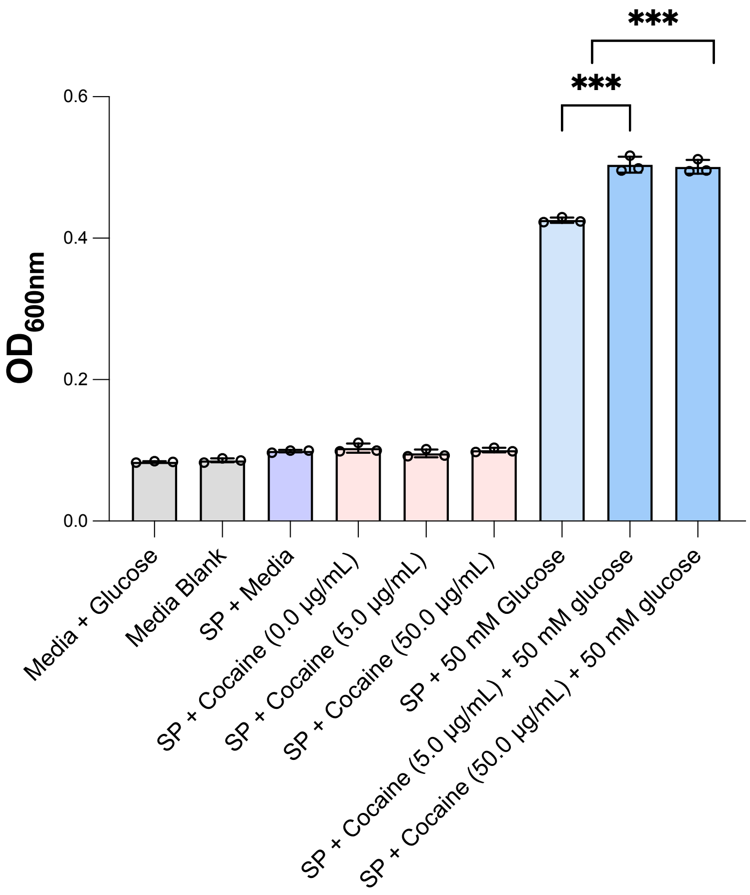

Supplemental Figure 3

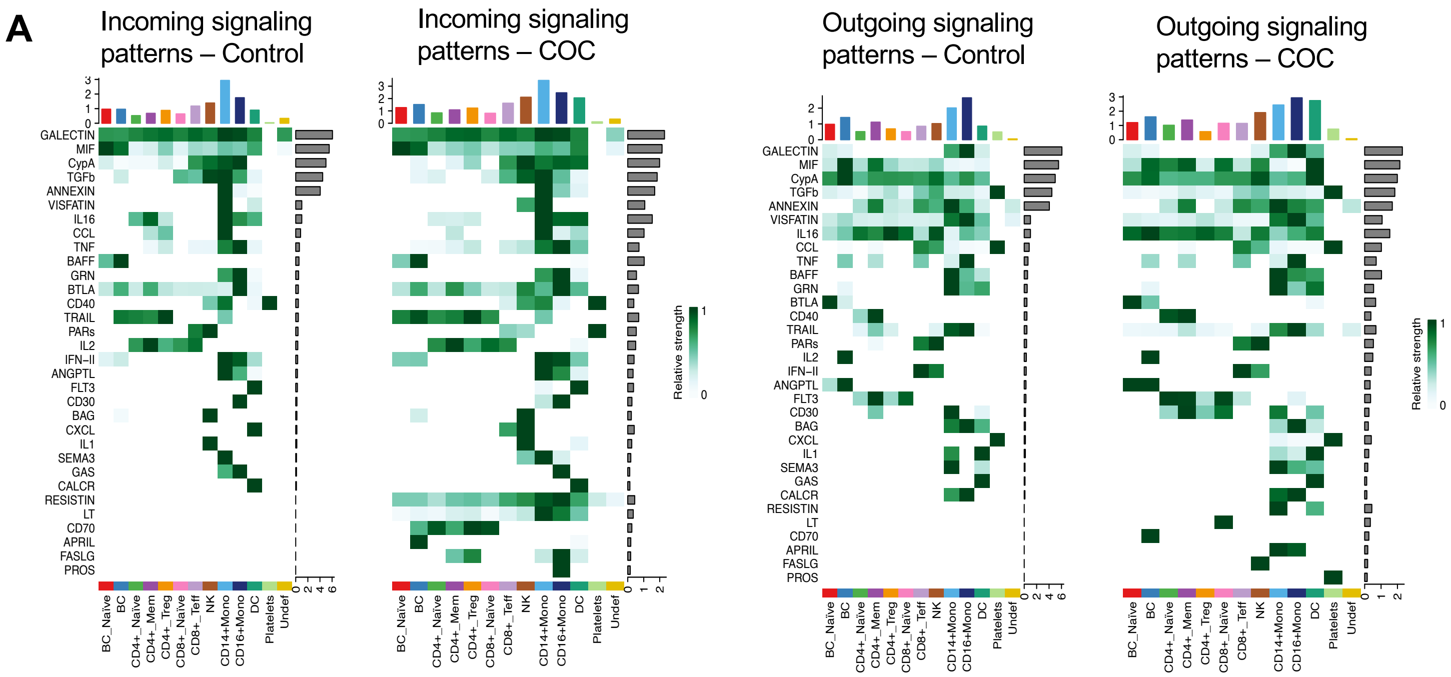

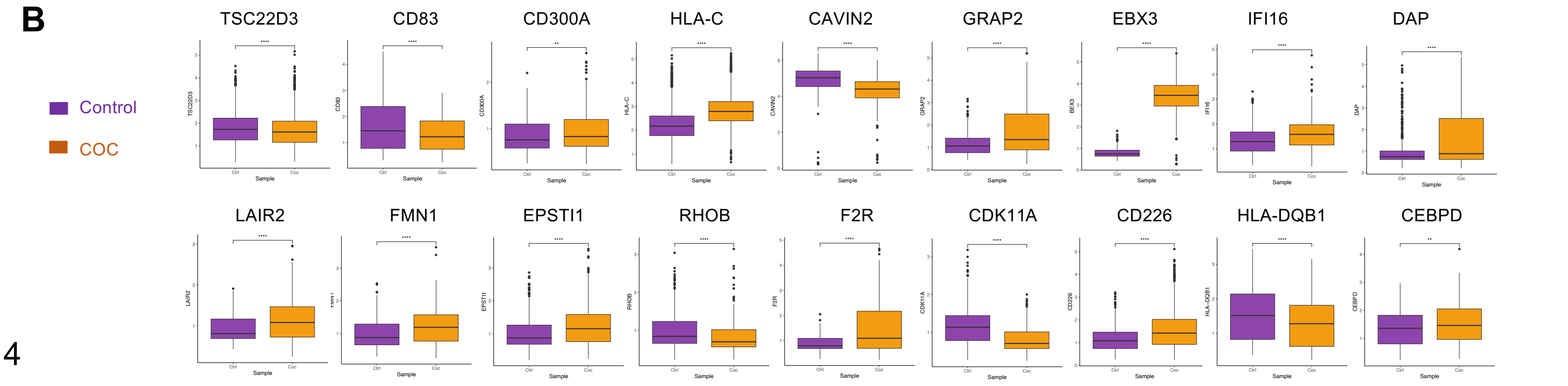

Supplemental Figure 4
